# Supplementary material for: PolyRapid: automated high-throughput screening of polymers using a computational workflow
Source: Soft Matter. 2026 Aug 12;22(33):5480–92. doi: 10.1039/d6sm00265j (PMC13464363; doi:10.1039/d6sm00265j)

Supplementary Information (SI) for Soft Matter.
This journal is © The Royal Society of Chemistry 2026

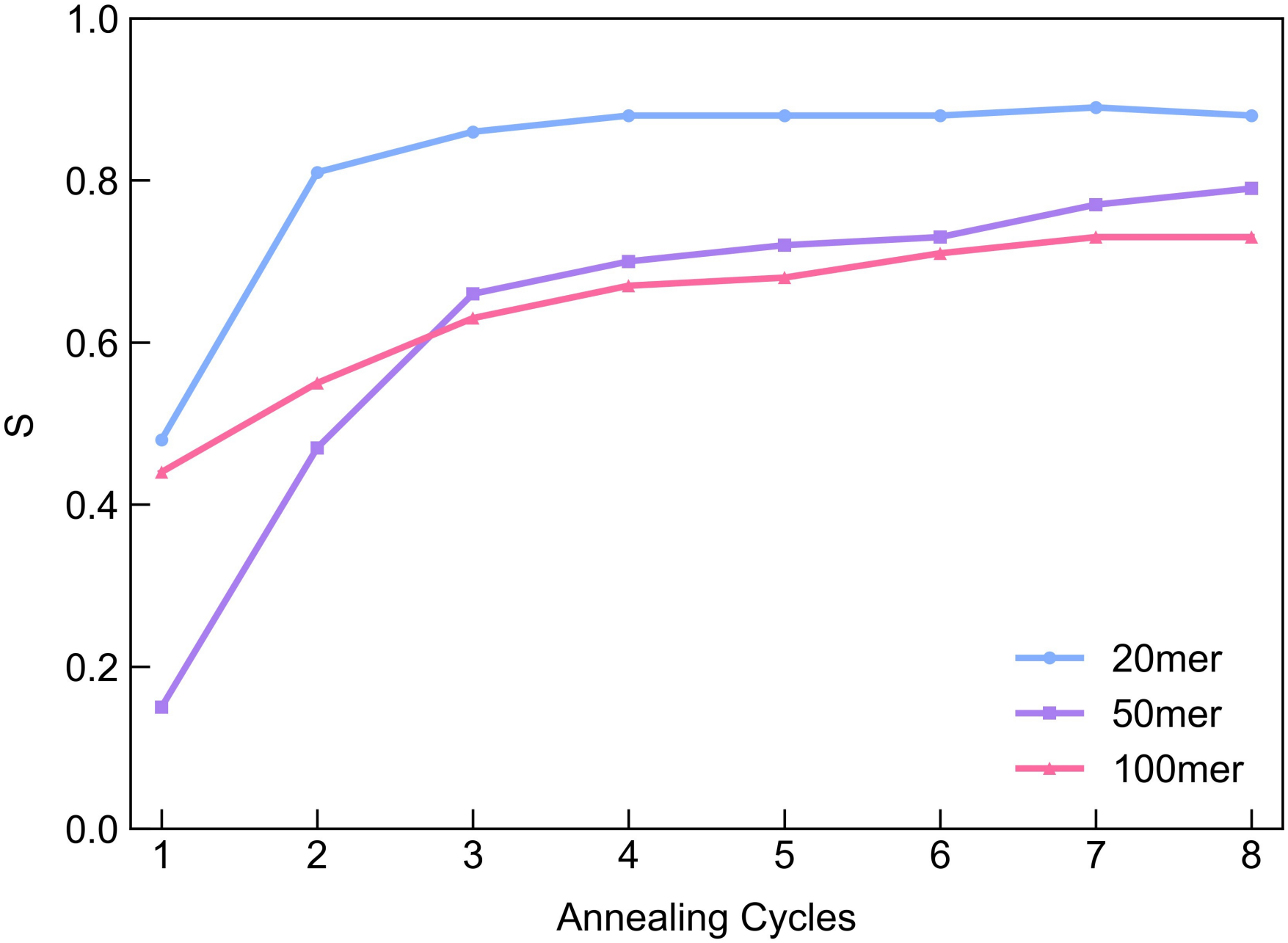

Supplement: Supplementary file 4 [file SM-022-D6SM00265J-s005.pdf]
