# Supplementary material for: PolyRapid: automated high-throughput screening of polymers using a computational workflow
Source: Soft Matter. 2026 Aug 12;22(33):5480–92. doi: 10.1039/d6sm00265j (PMC13464363; doi:10.1039/d6sm00265j)
Supplement: Supplementary file 5 [file SM-022-D6SM00265J-s006.pdf]

Supplementary Information (SI) for Soft Matter.
This journal is © The Royal Society of Chemistry 2026

Supporting Information for:

PolyRapid: Automated High-Throughput Screening of Polymers Using a Computational Workflow

Lois Smith[a], Samuel Ericson[a], Vittoria Fantauzzo[b], Chin Yong[c], Paola Carbone[a,*], Alessandro Troisi[b,**]

[a] Department of Chemistry, University of Manchester, Oxford Road, Manchester, M13 9PL, UK

[b] Department of Chemistry, University of Liverpool, Liverpool L69 7ZD, U.K.

[c] Computational Materials and Molecular Science, STFC Daresbury Laboratory, Warrington WA4 4AD, UK

*Email: Paola.Carbone@manchester.ac.uk

**Email: a.troisi@liverpool.ac.uk

S.1 Polymer Dataset Details

Monomer SMILES were manually curated from handbooks [1-3] and online sources, [4-13] and homopolymers built from the repeat unit. The polymer dataset was chosen among those for which reproducible experimental data was available, and to cover a large variety of polymer families (polyolefins, acrylic polymers, polyvinyls, styrenics polyethers, polyesters, polyamides, diene elastomers, fluoropolymers). Homopolymer SMILES are reported in Table S.1. For brevity, we do not report the polymer name, but instead report the polymer ID (PID) which can be used to look up the polymer names, along with the other data calculated in this work (radius of gyration (R_g), end-to-end distance (R_{ee}) etc.) in the PolyRapid Github. We also report the computed and experimental densities (ρ_{MD} and ρ_{exp} respectively) in Table S.1, with the former being averaged over the final 5 ns of the last annealing cycle in the protocol, along with a brief description on discrepancies in temperature, crystallinity and tacticity between simulation and experiment where possible, if they are recorded in the experimental source. The data from our simulations is reported at 300 K with isotactic configurations where applicable. We determine the crystallinity using the nematic order parameter (S) (see Section S.3), for which we only distinguish between amorphous ($S < 0.1$) and non-amorphous ($S > 0.1$).

Table S.1: Polymer SMILES, PID, computed density (ρ_{MD}), standard deviation (σ_ρ), experimental density (ρ_{exp}), experimental source and a brief statement on discrepancy in temperature, crystallinity and tacticity between the experimental source and simulation conditions. In the statement we also include polymers where we could only locate a monomer density. In the case of the crystallinity, we note cases where we achieved $S < 0.1$ in simulation but the data source specifies 'crystalline' or 'semi-crystalline'.

SMILES	PID	ρ_{MD} (kg/m^3)	σ_ρ (kg/m^3)	ρ_{exp} (kg/m^3)	source	Conditions discrepancy
[*]CC[*]	W01_P001	838.0396	3.5159	857	[1]	298 K
[*]CC(Cl)[*]	W01_P002	1362.697	1.9435	1385	[1]	-
[*]CC(F)[*]	W01_P003	1217.42	2.5378	1360	[2]	Atactic
[*]CC(C)[*]	W01_P004	841.4272	2.0614	853	[2]	298 K
[*]CC=CC[*]	W01_P005	875.4781	2.0844	950	[2]	-

[*]CC(CC)[*]	W01_P006	840.4003	2.1853	859	[2]	296 K
[*]C(F)(F)C[*]	W01_P007	1492.386	4.1597	1680	[2]	-
[*]C(Cl)(Cl)C[*]	W01_P008	1687.381	1.7497	1775	[2]	-
[*]CC(C)(C(=O)OCc1ccccc1)[*]	W01_P009	1131.18	1.873	1179	[1]	<5% isotactic
[*]CC(OCC)[*]	W01_P010	942.8733	2.2199	968	[1]	-
[*]CC(C#N)[*]	W01_P011	1081.019	1.2686	1155	[1]	-
[*]CC(C(=O)N(C)C)[*]	W01_P012	1055.97	1.7072	962	[4]	273 K, Monomer
[*]CC([*])C1=CC=CC=C1	W01_P013	1006.41	1.7874	1053	[2]	Atactic
[*]CCO[*]	W01_P014	1097.659	2.2138	1127	[1]	-
[*]C/C=C(C)/C[*]	W01_P015	881.0795	1.7967	960	[2]	-
[*]CC(O)[*]	W01_P016	1212.289	1.2956	1269	[1]	-
[*]C(=O)CCCCC(=O)NCCCCCCN[*]	W01_P017	1067.231	1.5205	1080	[1]	-
[*]CC(C(=O)N)[*]	W01_P018	1301.637	1.4464	1302	[5]	298 K
[*]CC(C)(C(=O)OC)[*]	W01_P019	1123.399	1.6802	1170	[1]	atactic
[*]CC(C(=O)OC)[*]	W01_P020	1181.295	1.9061	1220	[1]	298 K
[*]CC(C)(C(=O)O)[*]	W01_P021	1259.885	1.3755	1285	[2]	298 K, atactic
[*]CC(C(=O)O)[*]	W01_P022	1353.926	1.4241	1220	[2]	-
[*]C(OCCCCCCCC)C[*]	W01_P023	884.515	2.1104	914	[1]	-
[*]C(OCCC)C[*]	W01_P024	921.0367	2.1954	937	[1]	-
[*]CC(C(=O)OCC)[*]	W01_P025	1107.916	2.1983	1120	[1]	-
[*]CC(N)[*]	W01_P026	1147.079	1.8182	1150	[6]	
[*]CC(OC)[*]	W01_P027	999.6397	2.4097	1037	[1]	-
[*]OCCCCOC(=O)c1ccc(C(=O)[*])cc1	W01_P028	1211.755	1.8815	1256	[1]	-
[*]CC(N1CCCC1=O)[*]	W01_P029	1090.863	1.7858	1250	[1]	298 K
[*]CC(N1C2=C(C=CC=C2)C2=C1C=CC=C2)[*]	W01_P030	1095.049	1.7047	1184	[1]	-
[*]CC(C1=CC=CC=N1)[*]	W01_P031	1063.798	1.6621	1153	[7]	-
[*]C(OC(=O)CC)C[*]	W01_P032	1095.575	2.0534	1020	[8]	298 K
[*]CC(OC(C)=O)[*]	W01_P033	1144.243	1.8235	1190	[2]	298 K
[*]CC(C(=O)OCCCC)[*]	W01_P034	1032.841	2.0557	1080	[1]	-
[*]C(c1ccc(F)cc1)C[*]	W01_P035	1126.142	2.1704	1100.5	[1]	-
[*]C(c1c(Cl)cccc1)C[*]	W01_P036	1192.954	1.8861	1250	[1]	298 K
[*]C(c1cc(Cl)ccc1)C[*]	W01_P037	1184.46	1.8928	1241	[1]	298 K
[*]C(c1ccc(Cl)cc1)C[*]	W01_P038	1181.782	1.8929	1237.5	[1]	298 K
[*]CC(C(=O)OCCC)[*]	W01_P039	1066.412	2.248	1100	[1]	-

[*]CC1=CC=C(C[*])C=C1	W01_P040	1072.955	1.4306	1100	[9]	-
[*]C(C(=O)C)C[*]	W01_P041	1087.187	1.7299	1168	[1]	-
[*]C(NC(=O)C)C[*]	W01_P042	1115.191	1.5917	1060	[10]	-
*C(C)C(*)C(C)=C	W01_P043	862.2799	1.6721	850	[1]	298 K
[*]C(C)O[*]	W01_P044	1040.035	2.2115	1140	[1]	-
[*]OCCOC(=O)c1ccc(C(=O)O[*])cc1	W01_P045	1318.132	1.6598	1333	[1]	-
[*]OCCCCCCCCCCOC(=O)c1ccc(C(=O)O[*])cc1	W01_P046	1110.978	1.8658	1102.5	[1]	-
[*]OCCCCCCCCCCOC(=O)CCCCCCCCC(=O)[*]	W01_P047	992.9235	2.1009	1086	[1]	298 K
[*]OCCCCCCCCCCOC(=O)CCCCC(=O)[*]	W01_P048	1028.625	2.0109	973	[1]	-
C1CC(C[*])CCC1COC(=O)c1ccc(C(=O)O[*])cc1	W01_P049	1141.756	1.6817	1190	[1]	-
C1CC(CO[*])CCC1COC(=O)c1ccc(C(=O)O[*])cc1	W01_P050	1169.831	1.5577	1195	[1]	-
[*]C(c1ccc(C)cc1)C[*]	W01_P051	967.6525	1.9475	1019	[1]	298 K
[*]C(=O)CCN[*]	W01_P052	1240.767	1.5086	1330	[2]	298 K, drawn fibers
[*]CC(OCCCC)[*]	W01_P053	908.7416	2.2891	926	[1]	-
[*]CC(OCC(C)C)[*]	W01_P054	901.3483	1.9702	923	[1]	isotactic (S)-form
[*]C(=O)CCCCCO[*]	W01_P055	1083.36	2.2504	1147	[2]	Semi-crystalline
[*]C/C=C(C)\C[*]	W01_P056	880.2133	2.2639	913	[2]	-
c1cc([*])ccc1O[*]	W01_P057	1225.058	1.8159	1270	[2]	-
[*]C(c1ccc(O)cc1)C[*]	W01_P058	1124.129	1.6095	1160	[11]	298 K
CCCCCCCCCOC(=O)C([*])(C)C[*]	W01_P059	960.4907	2.1879	971	[1]	298 K
[*]CO[*]	W01_P060	1291.193	1.9381	1250	[1]	-
[*]C(c1ccc(OC)cc1)C[*]	W01_P061	1065.222	1.9745	1118	[1]	-
CCCCCOC(=O)C([*])(C)C([*])	W01_P062	1000.793	1.9419	1032	[1]	-
[*]C1=CC=C(C=C[*])C=C1	W01_P063	1113.304	1.6671	1283	[1]	-
[*]OCC(O)(C1CC(O)C(C[*])CC1)	W01_P064	1134.827	1.5295	1090	[1]	-
[*]C(OCOc1ccccc1)C[*]	W01_P065	1158.09	1.9197	1198	[1]	293 K
[*]C(C1CCCCC1)C[*]	W01_P066	882.8946	1.6869	946	[1]	298 K
[*]C(C1CCCC1)C[*]	W01_P067	915.5869	1.8224	965	[1]	-
[*]C(C1CC1)C[*]	W01_P068	891.979	1.834	975	[1]	-
[*]CC(O)COCCCOC(O)CO[*]	W01_P069	1193.491	1.778	1050	[1]	

[*]C(=O)c1ccc(C(=O)NCCCCCCN[*])cc1	W01_P070	1150.585	1.4936	1210	[1]	293 K
C1=CC(=CC=C1[*])S(=O)(=O)C2=CC=C(C=C2)O[*]	W01_P071	1329.73	1.7978	1370	[1]	-
[*]C1=CC=C(C=C1)S(=O)(=O)C2=CC=C(C=C2)OC3=CC=C(C=C3)C4=CC=C(C=C4)O[*]	W01_P072	1249.767	1.8135	1290	[1]	-
[*]c1ccc(S(=O)(=O)c2ccc(Oc3ccc(C(C)(C)c4ccc(O[*])cc4)cc3)cc2)cc1	W01_P073	1202.299	1.7199	1240	[1]	-
[*]Nc1ccc(Nc2ccc(Nc3ccc(Nc4ccc(C[*])cc4)cc3)cc2)cc1	W01_P074	1186.245	1.6065	1360	[14]	298 K
[*]NCCCCCCCCCCC(=O)[*]	W01_P075	968.4281	1.8647	1010	[2]	Up to 25% crystalline
[*]C(C)C(=O)O[*]	W01_P076	1214.481	1.7086	1248	[2]	-
[*]CC(C(=O)NC(C)C)[*]	W01_P077	1010.543	1.7338	1070	[1]	-
[*]CC(OC(C)C)[*]	W01_P078	927.1731	2.1123	925	[1]	298 K
[*]C1C(O1)COC2=CC=CC=C2[*]	W01_P079	1177.343	1.8746	1109	[12]	298 K
*CC(*)(CCCCCCCCCCCC)	W01_P080	842.763	1.8736	848	[1]	298 K
NCc1ccccc1CNC(=O)CCCCCCCCC(=O)	W01_P081	1086.696	1.5089	1140	[1]	-
[*]CC(C(=O)C)[*]	W01_P082	1081.212	1.8005	1168	[1]	298 K
[*]OC(C)CC(=O)[*]	W01_P083	1163.464	2.2834	1177	[2]	298 K
[*]C1CCC(CC[*])C1	W01_P084	925.3786	1.696	960	[13]	298 K
c1cc(O[*])ccc1C(=O)c2ccc(Oc3ccc([*])cc3)cc2	W01_P085	1218.422	1.8956	1264	[2]	-
[*]CCC(=O)[*]	W01_P086	1182.516	1.8824	1240	[1]	-
[*]C(=O)CCCCCCCCCCC(=O)NCCCCCCN[*]	W01_P087	996.5369	1.8231	1060	[1]	-
O=C(O[*])C1=CC(C=CC(C(OCC[*])=O)=C2)=C2C=C1	W01_P088	1259.985	1.7032	1340	[1]	273-298 K
[*]C(C1=CC=C(C(NC2=CC=C(N[*])C=C2)=O)C=C1)=O	W01_P089	1290.254	1.5217	1480	[1]	Crystalline
[*]CC([*])CC(C)C	W01_P090	828.7316	2.032	833	[1]	297 K
[*]CC(CCCCCCCCC)[*]	W01_P091	840.0535	1.9273	847	[1]	303 K
[*]CC(c1ccccc1)O[*]	W01_P092	1105.753	1.8929	1134	[1]	298 K
[*]OCCC[*]	W01_P093	1007.982	2.5545	982	[3]	-
[*]C(=O)CCCCC(=O)NCCCCN[*]	W01_P094	1108.917	1.513	1180	[1]	-
[*]C(=O)CCCCCCCCCCCCN[*]	W01_P095	960.778	2.1021	840	[2]	-
[*]CC(CC(C)(C)(C))	W01_P096	822.1596	1.8829	900	[1]	Crystalline

[*]						
[*]CC(Cl)=CC[*]	W01_P097	1213.257	2.1311	1230	[1]	-
[*]OCCCCOC(=O)CCC(=O)[*]	W01_P098	1203.178	2.2562	1260	[1]	-
[*]CC(CCCC)[*]	W01_P099	833.8054	1.854	860	[1]	293 K
[*]CC(CCCC)O[*]	W01_P100	912.7678	2.2412	947.5	[1]	-
[*]OCC(COC)[*]	W01_P101	1082.528	2.1739	1095	[1]	-
[*]CC(OCCCCCC)[*]	W01_P102	894.7144	2.1954	902	[1]	303 K
[*]CC(CCCCC)[*]	W01_P103	835.0498	1.8911	863	[1]	303 K

In Table S2, we further report the polymer SMILES, PID, experimental glass transition temperature (T_g^{exp}), and the corresponding experimental source (see Section S.8 for more details).

Table S2: Polymer SMILES, PID, computed glass transition temperature (T_g^{MD}), error estimate, experimental glass transition temperature (T_g^{exp}) and experimental source. Cases with unavailable experimental data are noted with 'N/A'.

SMILES	PID	T_g^{MD} (K)	*Err.* T_g^{MD} *(K)*	T_g^{exp} (K)	Source
[*]CC[*]	W01_P001	232.0342	0.2655	153	[1]
[*]CC(Cl)[*]	W01_P002	341.4533	0.3339	354	[1]
[*]CC(F)[*]	W01_P003	306.4224	0.2509	255.5	[2]
[*]CC(C)[*]	W01_P004	264.8332	0.2912	279.6	[2]
[*]CC=CC[*]	W01_P005	227.2812	0.2321	168.5	[2]
[*]CC(CC)[*]	W01_P006	267.4567	0.3202	252	[2]
[*]C(F)(F)C[*]	W01_P007	364.2348	0.2120	238	[2]
[*]C(Cl)(Cl)C[*]	W01_P008	411.3345	0.3520	255	[2]
[*]CC(C)(C(=O)OCc1ccccc1)[*]	W01_P009	339.9684	0.5155	327	[1]
[*]CC(OCC)[*]	W01_P010	285.7711	0.3976	235	[1]
[*]CC(C#N)[*]	W01_P011	415.0074	0.3690	360.75	[1]
[*]CC(C(=O)N(C)C)[*]	W01_P012	430.2430	0.4619	362	[4]
[*]CC([*])C1=CC=CC=C1	W01_P013	375.0410	0.5056	373	[2]
[*]CCO[*]	W01_P014	255.9936	0.2254	218	[1]
[*]C/C=C(C)/C[*]	W01_P015	261.1162	0.2846	208	[2]
[*]CC(O)[*]	W01_P016	397.5807	0.2789	353	[1]
[*]C(=O)CCCCC(=O)NCCCCCCN[*]	W01_P017	277.1023	0.4449	323	[1]
[*]CC(C(=O)N)[*]	W01_P018	430.3886	0.6376	461	[2]
[*]CC(C)(C(=O)OC)[	W01_P019	439.5537	0.3310	317	[1]

*]					
[*]CC(C(=O)OC)[*]	W01_P020	317.6401	0.2953	293.5	[1]
[*]CC(C)(C(=O)O)[*]	W01_P021	513.1509	0.5840	430.5	[2]
[*]CC(C(=O)O)[*]	W01_P022	415.2124	0.4040	387.5	[2]
[*]C(OCCCCCCCC)C[*]	W01_P023	215.5355	0.2673	194	[1]
[*]C(OCCC)C[*]	W01_P024	234.2356	0.3151	224	[1]
[*]CC(C(=O)OCC)[*]	W01_P025	245.0388	0.4718	248	[1]
[*]CC(N)[*]	W01_P026	-	-	N/A	N/A
[*]CC(OC)[*]	W01_P027	320.2076	0.3097	251	[1]
[*]OCCCCOC(=O)c1ccc(C(=O)[*])cc1	W01_P028	344.9245	0.3839	323	[1]
[*]CC(N1CCCC1=O)[*]	W01_P029	506.9349	0.6292	448	[1]
[*]CC(N1C2=C(C=CC=C2)C2=C1C=CC=C2)[*]	W01_P030	408.5201	0.9520	500	[1]
[*]CC(C1=CC=CC=N1)[*]	W01_P031	407.6056	0.6084	357	[1]
[*]C(OC(=O)CC)C[*]	W01_P032	187.9326	0.4097	283	[1]
[*]CC(OC(C)=O)[*]	W01_P033	385.6957	0.3654	305	[2]
[*]CC(C(=O)OCCCC)[*]	W01_P034	248.0818	0.3119	230	[1]
[*]C(c1ccc(F)cc1)C[*]	W01_P035	410.8244	0.4473	368	[1]
[*]C(c1c(Cl)cccc1)C[*]	W01_P036	379.6637	0.5603	392	[1]
[*]C(c1cc(Cl)ccc1)C[*]	W01_P037	415.8529	0.4968	363	[1]
[*]C(c1ccc(Cl)cc1)C[*]	W01_P038	363.3831	0.7328	402	[1]
[*]CC(C(=O)OCCC)[*]	W01_P039	220.4800	0.3529	228	[15]
[*]CC1=CC=C(C[*])C=C1	W01_P040	361.0922	0.5154	319.5	[9]
[*]C(C(=O)C)C[*]	W01_P041	372.8958	0.3725	308	[1]
[*]C(NC(=O)C)C[*]	W01_P042	-	-	N/A	N/A
*C(C)C(*)C(C)=C	W01_P043	444.3686	0.6976	276	[1]
[*]C(C)O[*]	W01_P044	300.5621	0.3405	243	[1]
[*]OCCOC(=O)c1ccc(C(=O)O[*])cc1	W01_P045	343.8326	0.5142	340	[1]
[*]OCCCCCCCCCCOC(=O)c1ccc(C(=O)O[*])cc1	W01_P046	292.6640	0.3734	281	[1]
[*]OCCCCCCCCCCOC(=O)CCCCCCCCC(=O)[*]	W01_P047	243.1888	0.2839	209	[1]
[*]OCCCCCCCCCCOC(=O)CCCCC(=O)[*]	W01_P048	244.8819	0.2524	199	[1]
C1CC(C[*])CCC1COC(=O)c1ccc(C(=O)O[*])cc1	W01_P049	373.8530	0.5611	365	[1]

C1CC(CO[*])CCC1COC(=O)c1ccc(C(=O)O[*])cc1	W01_P050	366.7308	0.4548	363	[1]
[*]C(c1ccc(C)cc1)C[*]	W01_P051	431.6076	0.5049	382	[1]
[*]C(=O)CCN[*]	W01_P052	358.5976	0.4116	418.5	[2]
[*]CC(OCCCC)[*]	W01_P053	230.2949	0.3048	218.5	[1]
[*]CC(OCC(C)C)[*]	W01_P054	273.1558	0.3853	251.5	[1]
[*]C(=O)CCCCCO[*]	W01_P055	252.6062	0.2698	201	[2]
[*]C/C=C(C)\C[*]	W01_P056	271.5849	0.2839	201.5	[2]
c1cc([*])ccc1O[*]	W01_P057	321.5138	0.4156	363	[2]
[*]C(c1ccc(O)cc1)C[*]	W01_P058	427.5756	0.4858	430.5	[11]
CCCCCCCCOC(=O)C([*])(C)C[*]	W01_P059	235.4613	0.3114	228	[1]
[*]CO[*]	W01_P060	301.5328	0.2156	225.5	[1]
[*]C(c1ccc(OC)cc1)C[*]	W01_P061	290.6064	0.5154	380	[1]
CCCCCOC(=O)C([*])(C)C([*])	W01_P062	256.8242	0.3933	300	[1]
[*]C1=CC=C(C=C[*])C=C1	W01_P063	393.7582	0.5021	281	[1]
[*]OCC(O)(C1CC(O)C(C[*])CC1)	W01_P064	-	-	N/A	N/A
[*]C(OCOc1ccccc1)C[*]	W01_P065	326.3669	0.3639	344	[1]
[*]C(C1CCCCC1)C[*]	W01_P066	397.3175	0.6042	385	[1]
[*]C(C1CCCC1)C[*]	W01_P067	392.0086	0.5986	345.5	[1]
[*]C(C1CC1)C[*]	W01_P068	-	-	N/A	N/A
[*]CC(O)COCCCOC(O)CO[*]	W01_P069	325.3371	0.2739	297.5	[1]
[*]C(=O)c1ccc(C(=O)NCCCCCCN[*])cc1	W01_P070	320.7501	0.4183	413	[1]
C1=CC(=CC=C1[*])S(=O)(=O)C2=CC=C(C=C2)O[*]	W01_P071	364.4953	0.6144	493	[1]
[*]C1=CC=C(C=C1)S(=O)(=O)C2=CC=C(C=C2)OC3=CC=C(C=C3)C4=CC=C(C=C4)O[*]	W01_P072	381.4458	0.6319	493	[1]
[*]c1ccc(S(=O)(=O)c2ccc(Oc3ccc(C(C)(C)c4ccc(O[*])cc4)cc3)cc2)cc1	W01_P073	419.9621	0.5919	458	[1]
[*]Nc1ccc(Nc2ccc(Nc3ccc(Nc4ccc(C[*])cc4)cc3)cc2)cc1	W01_P074	406.7030	0.3614	474	[2]
[*]NCCCCCCCCCCC(=O)[*]	W01_P075	257.9222	0.3422	315	[2]
[*]C(C)C(=O)O[*]	W01_P076	372.1228	0.3405	330	[2]

[*]CC(C(=O)NC(C)C)[*]	W01_P077	327.3860	0.7042	407	[1]
[*]CC(OC(C)C)[*]	W01_P078	231.9472	0.4115	265.5	[2]
[*]C1C(O1)COC2=CC=CC=C2[*]	W01_P079	354.7688	0.4463	282	[1]
*CC(*)(CCCCCCCCCCCC)	W01_P080	228.2649	0.2893	262	[1]
NCc1ccccc1CNC(=O)CCCCCCCCC(=O)	W01_P081	312.4417	0.4647	388	[1]
[*]CC(C(=O)C)[*]	W01_P082	326.7882	0.4715	308	[1]
[*]OC(C)CC(=O)[*]	W01_P083	319.5225	0.2991	282	[1]
[*]C1CCC(CC[*])C1	W01_P084	290.4250	0.3954	308	[2]
c1cc(O[*])ccc1C(=O)c2ccc(Oc3ccc([*])cc3)cc2	W01_P085	388.3366	0.4799	410	[1]
[*]CCC(=O)[*]	W01_P086	-	-	N/A	N/A
[*]C(=O)CCCCCCCCCCC(=O)NCCCCCCN[*]	W01_P087	284.4932	0.3263	319	[1]
O=C(O[*])C1=CC(C=CC(C(OCC[*])=O)=C2)=C2C=C1	W01_P088	396.2618	0.6036	393	[1]
[*]C(C1=CC=C(C(NC2=CC=C(N[*])C=C2)=O)C=C1)=O	W01_P089	390.5279	0.7210	600	[1]
[*]CC([*])CC(C)C	W01_P090	275.4097	0.4088	298	[1]
[*]CC(CCCCCCCCC)[*]	W01_P091	218.2392	0.2906	239.5	[1]
[*]CC(c1ccccc1)O[*]	W01_P092	310.6359	0.4545	313	[1]
[*]OCCC[*]	W01_P093	272.4356	0.2320	193.5	[3]
[*]C(=O)CCCCC(=O)NCCCCN[*]	W01_P094	296.6291	0.4180	316	[1]
[*]C(=O)CCCCCCCCCCCN[*]	W01_P095	252.2704	0.3665	323	[2]
[*]CC(CC(C)(C)(C))[*]	W01_P096	377.2094	0.5627	463	[1]
[*]CC(Cl)=CC[*]	W01_P097	277.5270	0.2843	236	[1]
[*]OCCCCOC(=O)CCC(=O)[*]	W01_P098	246.4947	0.2861	241	[1]
[*]CC(CCCC)[*]	W01_P099	235.7869	0.3449	219	[1]
[*]CC(CCCC)O[*]	W01_P100	255.5598	0.3378	204	[1]
[*]OCC(COC)[*]	W01_P101	289.6251	0.2926	211	[1]
[*]CC(OCCCCCC)[*]	W01_P102	236.2172	0.3016	198	[1]
[*]CC(CCCCC)[*]	W01_P103	228.1526	0.3582	235	[1]

S.2 Annealing Protocol Conditions Analysis

In order to verify that the initial starting configuration of the polymers does not bias their final structure via the random packing seed, compression pressure or starting density, we calculate the chain center-of-mass root mean square displacement (RMSD), along with the end-to-end vector autocorrelation function ($C_{ee}(\tau)$). These analyses were performed over the initial ~2000 ps of the first annealing trajectory (150–2000 ps), during which the system temperature decreases from approximately 800 K to 760 K. This high-temperature annealing window is used to assess whether polymer chains undergo sufficient translational and conformational relaxation to lose memory of their initial packing configuration before subsequent equilibration.

The end-to-end vector autocorrelation function is defined as

$$C_{ee}(\tau) = \frac{\left\langle \frac{1}{N}\sum_{i=1}^{N} \boldsymbol{R}_{ee,i}(t_0) \cdot \boldsymbol{R}_{ee,i}(t_0+\tau) \right\rangle_{t_0}}{\left\langle \frac{1}{N}\sum_{i=1}^{N} \boldsymbol{R}_{ee,i}(t_0) \cdot \boldsymbol{R}_{ee,i}(t_0) \right\rangle_{t_0}} \qquad \textit{Eq. S1}$$

where $\boldsymbol{R}_{ee,i}(t)$ is the end-to-end vector of chain i at time t. $C_{ee}(\tau)$ measures the persistence of chain orientation by comparing the end-to-end vector at time t_0 and a later time, $t_0+\tau$. $C_{ee}(\tau)$ decays over time as the polymer loses orientational memory of

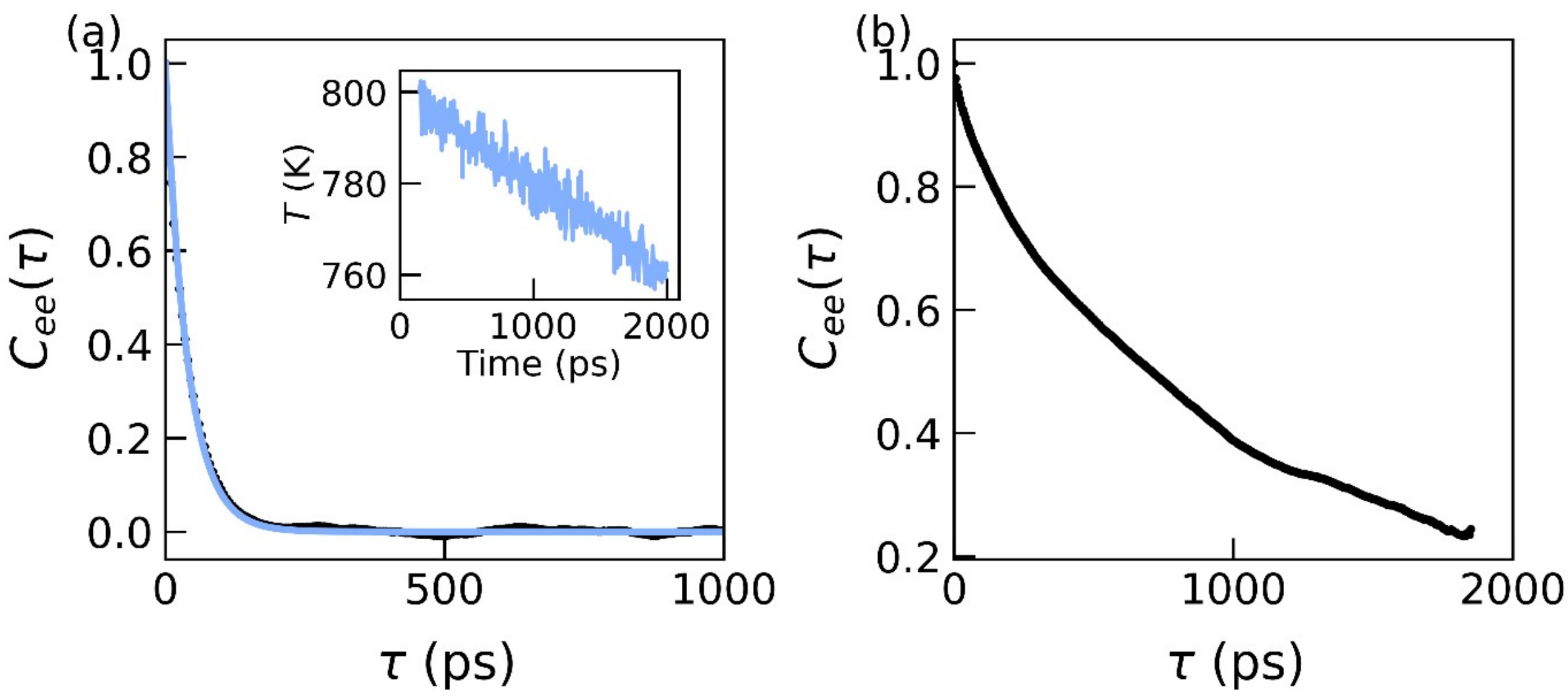

Figure S1. End-to-end vector autocorrelation function, $C_{ee}(\tau)$ for (a) polyethylene and (b) poly(vinyl cyclohexane). The inset shows the temperature range over which the data was taken. $C_{ee}(\tau)$ is truncated at 1000 ps for polyethylene.

its initial structure. $C_{ee}(\tau)$ for a more flexible polymer, polyethylene, and a more rigid polymer, poly(vinyl cyclohexane) are presented in Figure S1.

The RMSD in the 2 ns interval range varies from ~2 nm (poly(vinyl cyclohexane), $R_g =$ 0.963 nm) to ~9.5 nm (polyethylene, $R_g =$ 1.157 nm) suggesting sufficient thermal randomization and therefore independence from the initial box configuration and random seed. Furthermore, in polyethylene, the end-to-end vector autocorrelation function decays rapidly to approximately zero within 100-200 ps, indicating rapid loss of chain orientational memory. Fitting the exponential function $C_{ee}(\tau) = \exp(-\tau/\tau_c)$ (blue in Figure S1) provides a characteristic relaxation time of $\tau_c =$ 40 ps. In poly(vinyl cyclohexane), $C_{ee}(\tau)$ does not fully decorrelate during this time, indicating persistent orientational memory. Therefore, for additional scrutiny, we performed repeat simulations of poly(vinyl cyclohexane) with an alternate random packing seed (42), lower compression pressure (500 bar), higher compression pressure (1500 bar) and lower initial box density ($\rho = 9.6$ kg/m^3), and re-calculated ρ_{MD}, R_g and R_{ee}. The results are presented in Table S3, which also includes the percent change in these properties from the original simulation to the new tests.

Table S3: Structural metrics of poly(vinyl cyclohexane) post-equilibration for the original simulation and simulations with the listed changes. Note that the initial simulation had the following: random packing seed, 1000 bar compression pressure, ~26.3 kg/m^3 initial density. In brackets are the standard deviations and the percent change between the corresponding structural metric value and its initial value in the original simulation (% change = $|(X_{final} - X_{initial})| / X_{initial} \times 100$).

Simulation Details	Convergence steps num.	ρ_{MD} (kg/m^3)	R_g (nm)	R_{ee} (nm)
Original simulation	3	882.9 (1.7)	0.963 (0.003)	2.192 (0.02)
Random seed = 42	2	882.7 (1.8) (<0.1%)	0.964 (0.001) (0.1%)	2.222 (0.006) (1.5%)
500 bar pressure	3	884.1 (1.7) (0.14%)	0.943 (0.001) (2.1%)	2.225 (0.006) (1.8%)
1500 bar pressure	2	885.0 (1.9) (0.24%)	0.950 (0.001) (1.3%)	2.250 (0.006) (2.7%)
$\rho = 9.6$ kg/m^3 box	2	883.7 (1.6) (<0.1%)	0.945 (0.001) (1.9%)	2.202 (0.006) (0.5%)

Across all protocol conditions, the final bulk density varies by less than 0.3%, with all values remaining within approximately one standard deviation of the original simulation. Slightly larger variations are observed in R_g and R_{ee} (<3%), which is expected given that these quantities probe individual chain conformations rather than bulk properties. Overall, these results suggest that the final equilibrated structure is not strongly sensitive to the initial packing protocol.

Since the cooling rate and simulation box size may also affect the final equilibrated structure, additional tests were done using a faster cooling rate of 30 K/ns (original 20 K/ns) and smaller/larger systems containing approximately 20,000 and 100,000 atoms (original 50,000 atoms), respectively, while keeping the polymer chain length fixed at 20 repeat units. These tests were performed for the 8 polymers listed in Table S4, selected by sampling two polymers from each quartile of the computed glass transition temperature distribution in order to capture a range of polymer rigidities.

Table S4: Polymer name, SMILES and computed glass transition temperature (T_g^{MD}) of the 8 polymers used for the cooling rate and box size tests.

Polymer name	SMILES	T_g^{MD} (K)
Polyethylene	[*]CC[*]	232.0
Polyethylene oxide	[*]CCO[*]	256.0
Polypropylene	[*]CC(C)[*]	264.8
Poly(p-phenyl oxide)	c1cc([*])ccc1O[*]	321.5
Polystyrene	[*]CC([*])C1=CC=CC=C1	375.0
Parylene	[*]CC1=CC=C(C[*])C=C1	361.1
Poly vinyl cyclohexane	[*]C(C1CCCCC1)C[*]	397.3
Poly(4-vinylphenol)	[*]C(c1ccc(O)cc1)C[*]	427.6

Figure S2 presents the signed percent change in ρ_{MD}, R_g, and R_{ee} relative to the original simulation for each polymer across the additional test conditions.

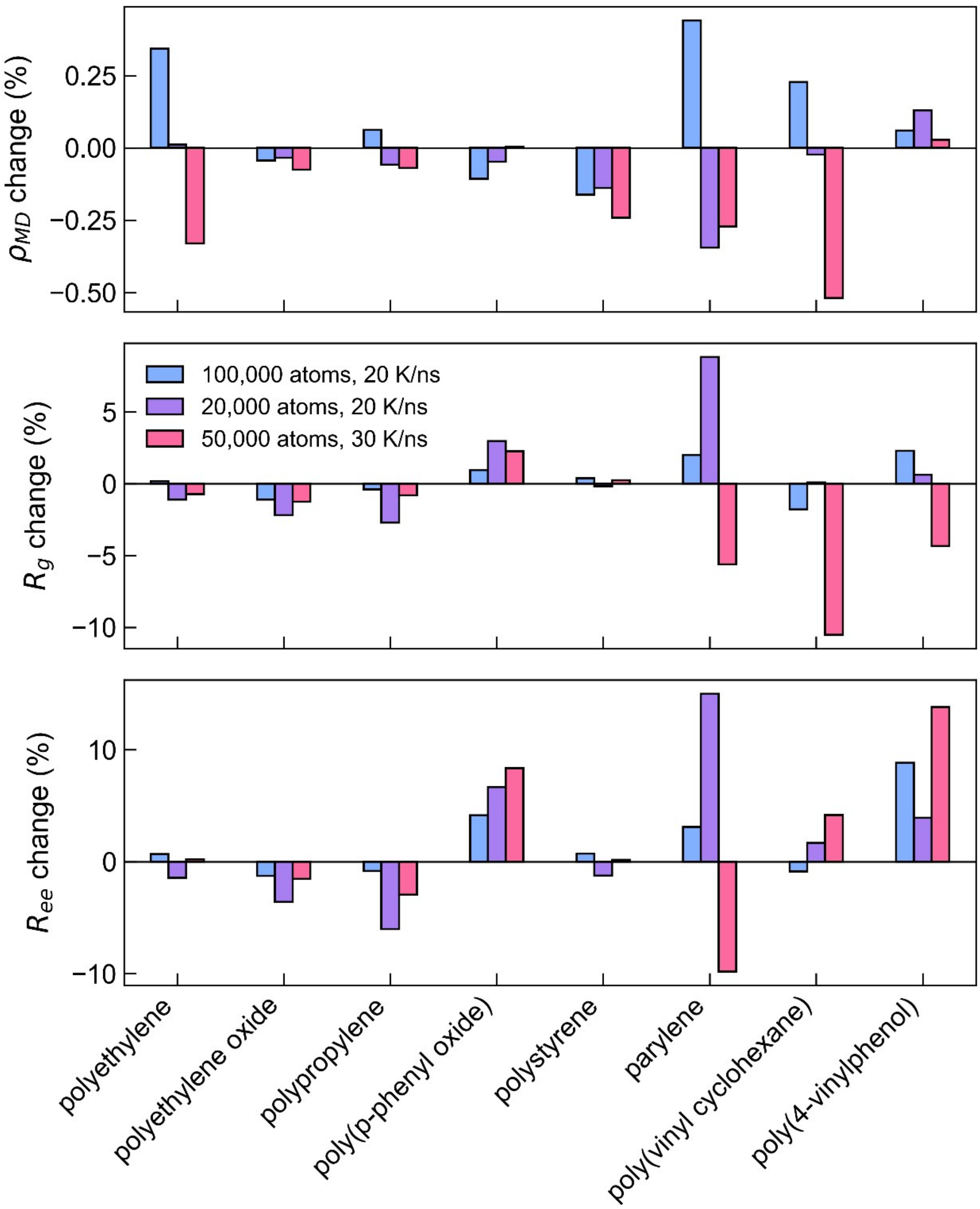

Figure S2: Percent deviation between the original ρ_{MD} (top), R_g (middle) and R_{ee} (bottom), and their values from simulations with 100,000 atoms (blue), 20,000 atoms (purple) and 30 K/ns cooling rate (pink), for the 8 polymers in Table S4.

Across all polymers and protocol variations, the final bulk density remains highly reproducible, with deviations of less than 0.5%, indicating that density is largely insensitive to cooling rate and box size under the conditions tested. For lower-T_g^{MD}

(more flexible) polymers, changes in R_g and R_{ee} are also generally small across all tests. In contrast, larger deviations in these conformational properties are observed for higher-T_g^{MD} (more rigid) polymers, particularly in the 20,000 atom and 30 K/ns tests. The relatively small deviations observed in the 100,000 atom simulations suggest that the standard 50,000 atom simulation cell is already sufficient for the structural properties considered here, whereas the 20,000 atom systems appear more susceptible to finite-size effects, particularly for rigid polymers. These results indicate that while density is robust across the tested conditions, additional care may be required when evaluating conformational properties such as R_g and R_{ee}, especially for rigid polymers under smaller box sizes or faster cooling rates.

S.3 Measure of Crystallinity

Crystallinity effects play a significant role in a polymer's physical properties, including density, and experimental data sources do not always clearly differentiate between amorphous and crystalline phases. This can introduce discrepancies when comparing with simulations that aim to have purely amorphous behaviour. For this reason, we have evaluated a nematic order parameter, *S*, computed from the largest eigenvalue of the orientational tensor, defined as

$$Q_{ab} = \frac{1}{N}\sum_{i=1}^{N}\left(\frac{3}{2}\mu_{i\alpha}\mu_{i\beta} - \frac{1}{2}\delta_{\alpha\beta}\right) \qquad \text{Eq. S1}$$

where $\mu_{i\alpha}$ represents the normalised axis of a monomer i ($\alpha, \beta = x,y,z$), which is defined by the axis corresponding to the smallest eigenvalue of the inertia tensor. N is the number of repeating units. The nematic order is then obtained as

$$S = \lambda_{max}(\mathrm{Q}) \qquad \text{Eq. S2}$$

with the largest eigenvalue of *Q* expected to fluctuate near zero for an amorphous substance, reflecting an absence of a preferred alignment along an axis. In contrast, a value approaching $S = 1$ indicates a strongly ordered, crystalline system. Following previous work[16], we define an amorphous polymer with $S < 0.1$. We compute the average *S* over the final 5 ns of the equilibration loop.

Figure S3 displays the distribution of S within our data, along with examples of polymers with increasing values of S which shows the corresponding increase in crystallinity.

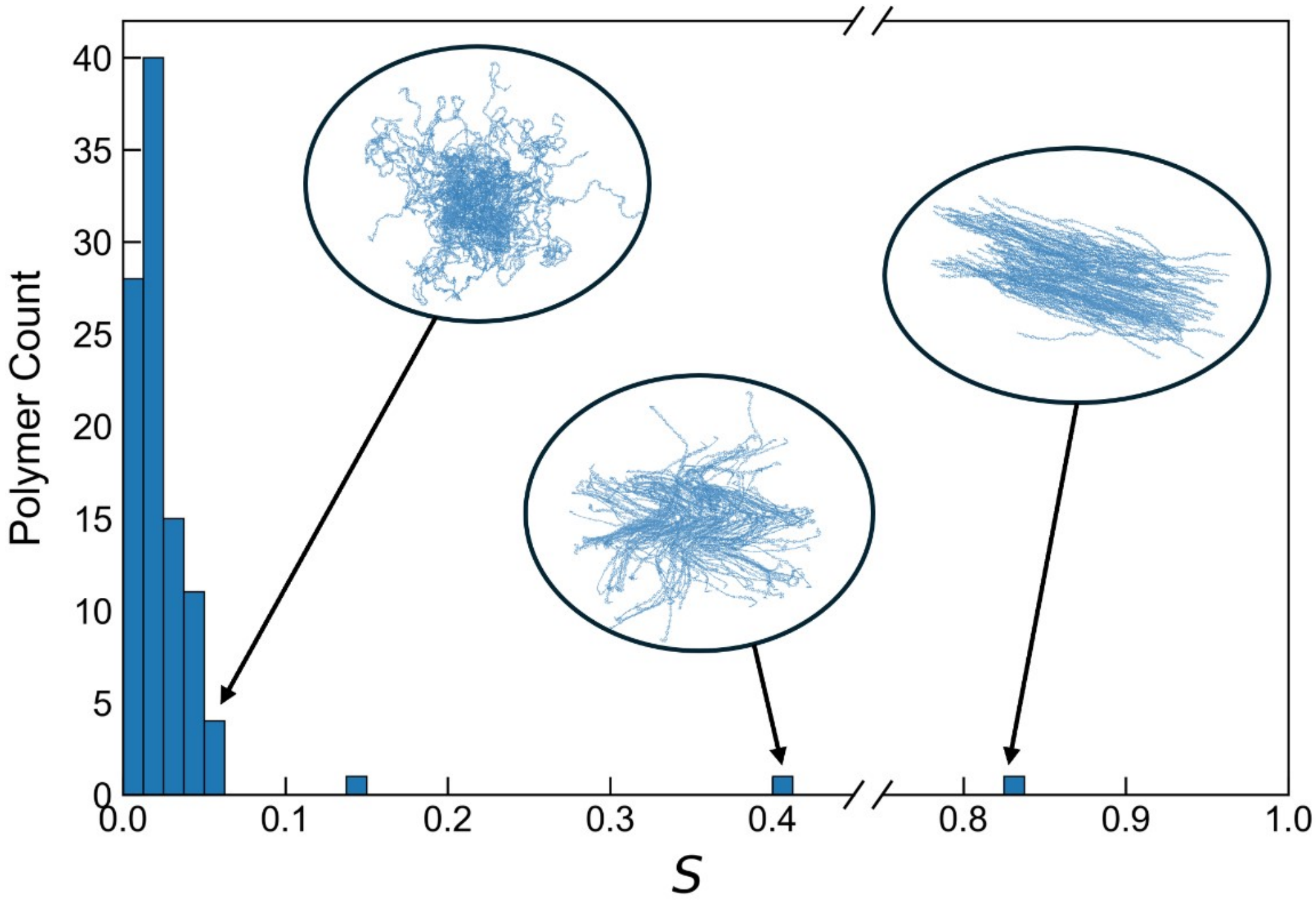

Figure S3. Distribution of the average nematic order parameter, S, across the last 5ns of the final annealing cycle of each polymer in the dataset. Non-equilibrated polymers were excluded from these results. The polymers depicted are: polyphenylsulfone ($S = 0.059$), Poly(p-phenylene vinylene) ($S = 0.40$) and Parylene ($S = 0.83$).

S.4 Annealing Protocol Convergence Metric Analysis

To assess the robustness of the equilibration protocol, we subjected 8 representative polymers, covering a range of chain rigidities, to additional annealing using a stricter threshold. See Table S1 in Section S.1 for a list of systems. Figure S4 shows an example polyethylene system comparing equilibration under the original RDF-criterion ($\Delta RDF_n < 0.02$) and the stricter criterion ($\Delta RDF_n < 0.008$) (see section 3.1 of the main text for definitions).

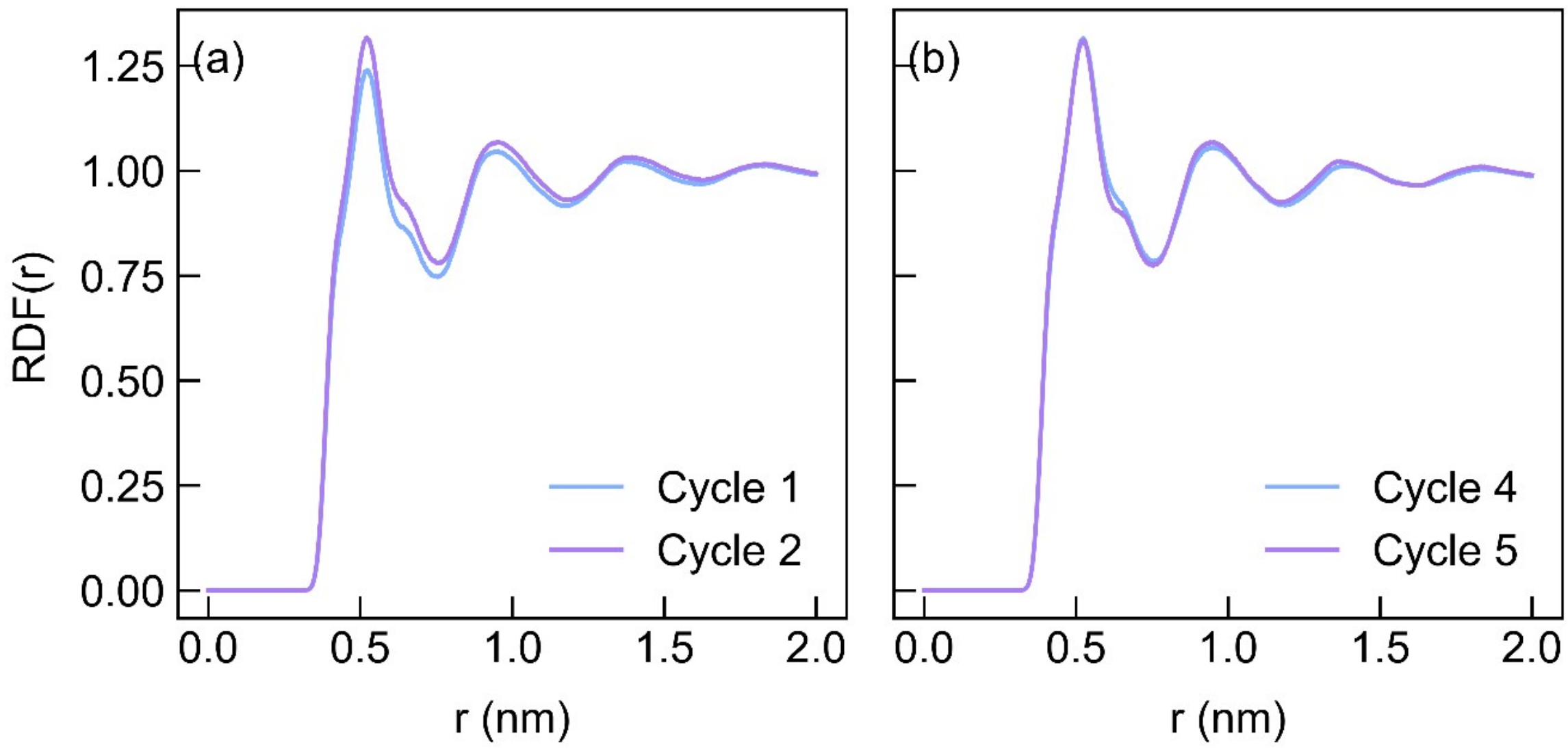

Figure S4: RDFs of polyethylene comparing equilibration under (a) the default convergence criterion ($\Delta RDF_n <$ 0.02 (actual $\Delta RDF_n =$ 0.0181)) and (b) a stricter criterion ($\Delta RDF_n <$ 0.008 (actual $\Delta RDF_n <$ 0.0069)). RDFs are extracted over the final 5 ns of the annealing cycle indicated by the cycle number in the figure legends.

As it can be seen from Figure S4(a), $\Delta RDF_n <$ 0.02 represents a very small change in polymer structure between annealing cycles, and for $\Delta RDF_n <$ 0.008 the change is visually indistinguishable. For each representative polymer, annealing was continued for a maximum of 6 cycles after initial equilibration. Simulations were halted earlier if $\Delta RDF_n <$ 0.008 was satisfied; otherwise, the final ΔRDF_n is reported.

Table S5 summarizes the polymers studied, the final ΔRDF_n values obtained, and the number of total annealing cycles performed. Every polymer reached $\Delta RDF_n <$ 0.008 in under 6 annealing cycles, except parylene which reached $\Delta RDF_n <$ 0.0089 in 8 annealing cycles. To test the effect of using alternative annealing metrics, we also investigate the density (ρ_{MD}), radius of gyration (R_g) and potential energy (PE), and report the number of annealing cycles required to achieve $\Delta\rho_{MD} < 0.2\%$, $\Delta R_g < 1\%$ and $\Delta PE < 1\%$, where Δ represents changes between consecutive annealing cycles. These thresholds are somewhat arbitrary but demonstrate that alternative equilibration metrics can lead to different estimates of equilibration times depending on the property

being probed. Note that poly(p-phenyl oxide) did not reach the prescribed ΔR_g threshold, and so its data was recorded after cycle 8 (ΔR_g~2.7%).

Table S5: Polymer name and number of cycles to reach equilibration by the metrics $\Delta RDF_n <$ 0.02, $\Delta RDF_n <$ 0.008, $\Delta\rho_{MD} < 0.2\%$, $\Delta R_g < 1\%$, and $\Delta PE < 1\%$.

Polymer Name	Cycle No. $\Delta RDF_n < 0.02$	Cycle No. $\Delta RDF_n < 0.0$	Cycle No. $\Delta\rho_{MD} < 0.2\%$	Cycle No. $\Delta R_g < 1\%$	Cycle No. $\Delta PE < 1\%$
Polyethylene	2	5	3	3	3
Polyethylene oxide	2	4	2	4	2
Polypropylene	3	5	2	2	2
Poly(p-phenyl oxide)	2	4	2	8	2
Polystyrene	2	4	2	2	2
Parylene	2	8	3	6	7
Poly(vinyl cyclohexane)	3	3	2	2	3
Poly(4-vinylphenol)	2	5	2	5	2

We further report the value of ρ_{MD}, R_g and PE for each polymer and annealing cycle in Figure S5.

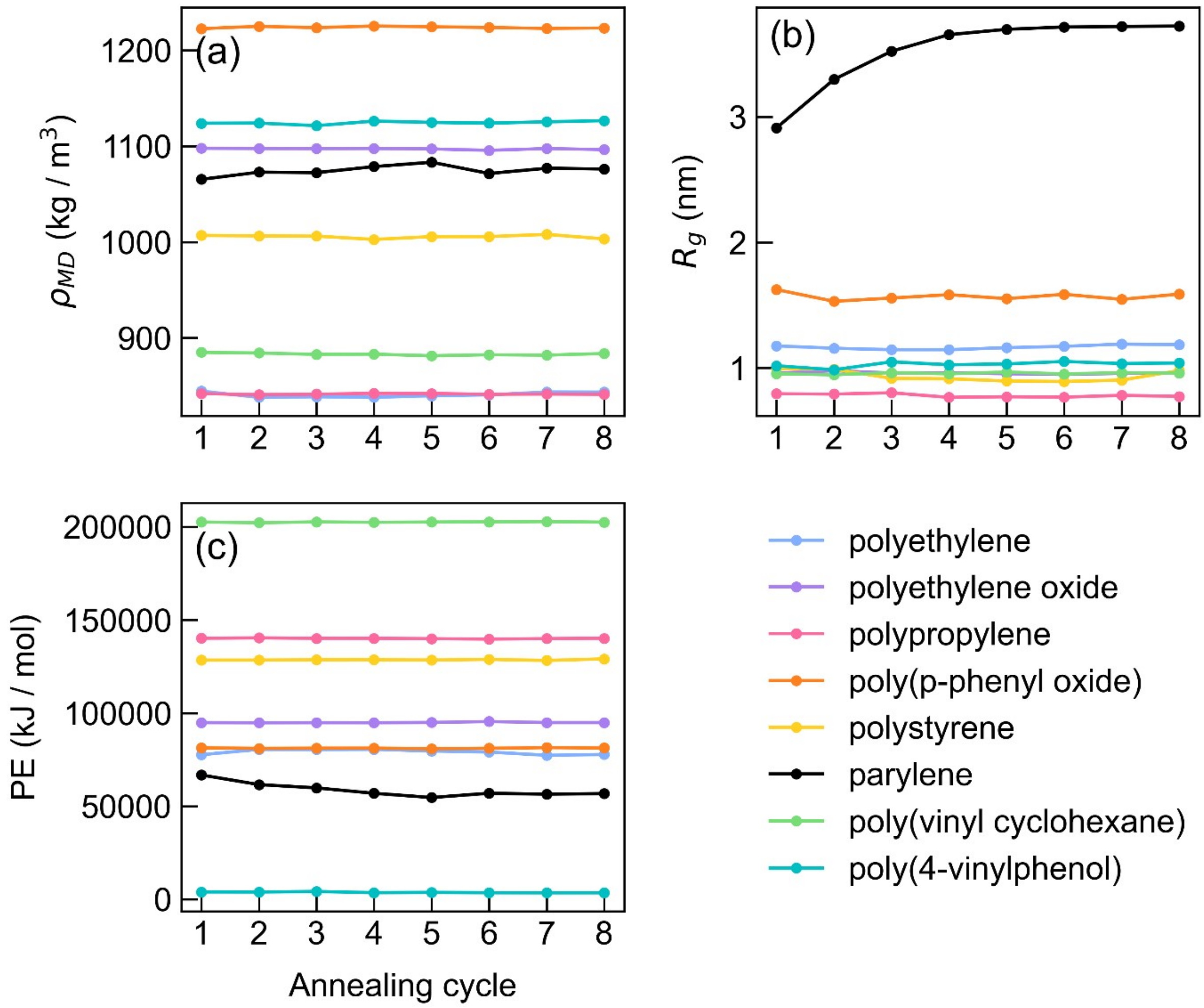

Figure S5: (a) ρ_{MD}, (b) R_g and (c) PE over 8 annealing cycles for the 8 polymers listed in Table S4. Values were averaged over the final 5 ns of each annealing cycle. Standard errors are too small to display.

Generally, $\Delta RDF_n <$ 0.02 and $\Delta\rho_{MD} < 0.2\%$ are the least stringent metrics for each polymer, and $\Delta RDF_n <$ 0.008 is the most stringent on average. The stringency of $\Delta R_g < 1\%$ and $\Delta PE < 1\%$ is highly system-dependent, with some polymers satisfying these thresholds rapidly and others requiring substantially more annealing cycles to do so. Notably, as shown in Figure S5, ρ_{MD}, R_g and PE remain relatively stable over the first eight annealing cycles for the majority of systems, suggesting that long equilibration times are not required for these properties. The exception is parylene,

which displays significant drift in R_g and PE, which is not captured using $\Delta RDF_n <$ 0.02. Considering this, since parylene was identified as one of the few non-amorphous polymers in our dataset based on its nematic order parameter, S, (see Section 3.2 of the main text and Section S.3 of the SI), we record its evolution, along with that of R_g, over the annealing cycles, in Figure S6(a). All quantities were averaged over the final 5 ns of their respective final annealing cycle. As a further test, because a global value of S cannot resolve local orientational order in systems containing multiple ordered domains, we calculated the distribution of local nematic order parameters (S_{local}) throughout the simulation box (Figure S6(b)). To probe local orientational order, we partitioned the simulation box into 64 equally sized cells of approximately $2 \times 2 \times$ 2 nm^2, where the cell length (~2 nm) corresponds to roughly 10 times the radius of gyration of a parylene monomer ($R_g \approx 0.1996$ nm), calculated from its monomer SMILES representation using RDKit. [17] Within each cell, S was calculated independently in every frame over the final 5 ns of annealing cycle 2 (satisfying $\Delta RDF_n <$ 0.02 between cycles 1 and 2) and annealing cycle 8 (satisfying $\Delta RDF_n <$ 0.01 between cycles 7 and 8), using the same procedure described in Section S.3, with monomers assigned to cells according to their center-of-mass positions. Histograms were then constructed from the resulting distributions of S_{local}. We additionally tested smaller cell sizes corresponding to approximately $5R_g$ and $3R_g$; while this affected the spatial resolution of local ordering, it did not alter the qualitative trend observed between cycles.

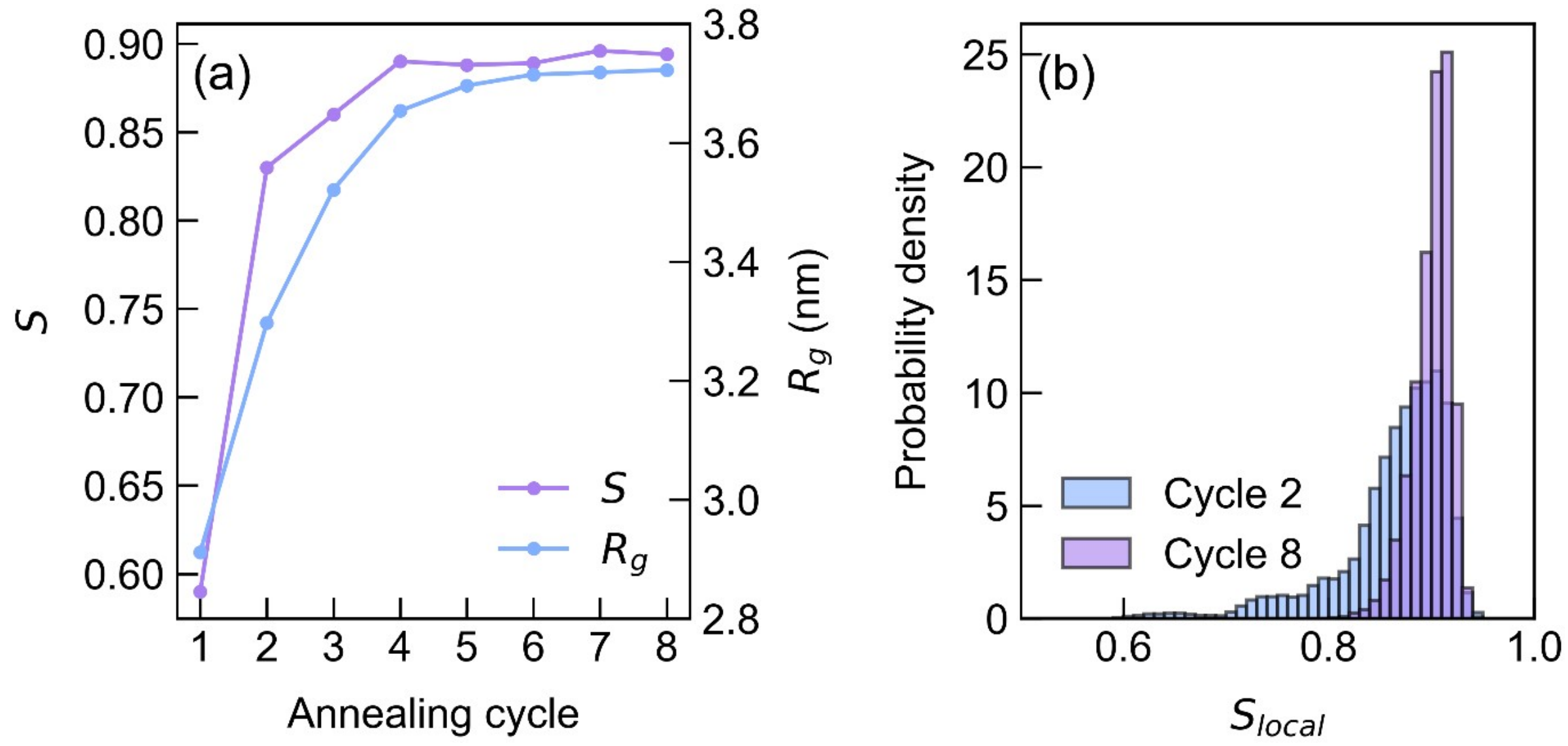

Figure S6: (a) Global nematic order parameter, S, and R_g against annealing cycle number for parylene (SMILES: [*]CC1=CC=C(C[*])C=C1). S and R_g standard errors are smaller than the plot point size. (b) Local order parameter, S_{local} distributions between annealing cycle 2 and 8.

The global nematic order parameter, S, and R_g both increase with annealing cycle number (Figure S6(a)), indicating progressive chain alignment and straightening during annealing. This trend eventually plateaus, suggesting that the system approaches a highly ordered crystalline state at later cycles. Consistent with this, the distribution of local nematic order parameters shifts to a narrower, more sharply peaked distribution between cycles 2 and 8, with the low-S_{local} tail present at cycle 2 largely disappearing by cycle 8 (Figure S6(b)). This indicates that locally disordered regions become less prevalent upon further annealing, consistent with increasing crystallinity.

ΔRDF_n measures short-range local structure and packing ($<$ 2nm), and is therefore less sensitive to subtle orientational rearrangements that do not substantially alter local intermolecular correlations. In contrast, relatively small changes in monomer alignment can produce measurable changes in R_g and a narrowing of the S_{local} distribution as locally disordered regions are reduced. Therefore, in very rigid polymers that display a high degree of orientational order, of which there are very few in our dataset (see Section S.3 of the SI), a single RDF-based convergence criterion may

not fully capture continued orientational refinement. As can be seen from Table S5, an alternative metric such as ΔR_g may improve the accuracy of our approach in such systems, whilst maintaining shorter equilibration times for amorphous polymers.

S.5 Procedure Computational Cost Breakdown

Table S6: Runtime distributions of selected systems run at target atom counts of 15,000 and 50,000 atoms utilising 80 cores running for a maximum of 2 equilibration cycles.

Polymer name	Exact atom count	Run time / h	Exact atom count	Run time / h
Polyethylene	14884	4.25	49898	12.00
Polyethylene oxide	14910	3.79	49984	9.87
Polypropylene	14924	4.28	49868	12.05
Poly(p-phenyl oxide)	14874	3.47	49950	9.38
Polystyrene	14812	4.47	49910	11.69
Parylene	14812	3.81	49910	9.84
Poly vinyl cyclohexane	14586	4.79	49946	12.36
Poly(4-vinylphenol)	14706	4.59	49932	11.73

Table S6 shows that the polymer with the largest monomer, Poly(4-vinylphenol) with 9 heavy atoms has a runtime within 1 standard deviation of the polymer with the smallest monomer, polyethylene, with 2 heavy atoms. This effect is also observed to be insignificant with regards to the chain stiffness with Parylene also falling within the same runtime range.

This also shows that the implementation of the adaptive equilibration protocol displays a predictable runtime distribution across the number of atoms per system making the process convenient for large scale screening of polymer systems.

S.6 Chain length convergence tests

To assess whether the equilibration protocol is suitable for higher molecular weight polymers, additional simulations were performed for 20-, 50-, and 100mer chains while maintaining a constant system size of ~100,000 atoms. The same eight representative polymers spanning a range of backbone rigidities, listed in Table S4, were used for this analysis. All systems were initialized in a $32 \times 32 \times 32\ \mathrm{nm}^3$ simulation box, and properties were averaged over the final 5 ns of the last annealing cycle after the structural convergence criterion $\Delta RDF_n <$ 0.02 was satisfied. In Figure S7, we present the density results (ρ_{MD}).

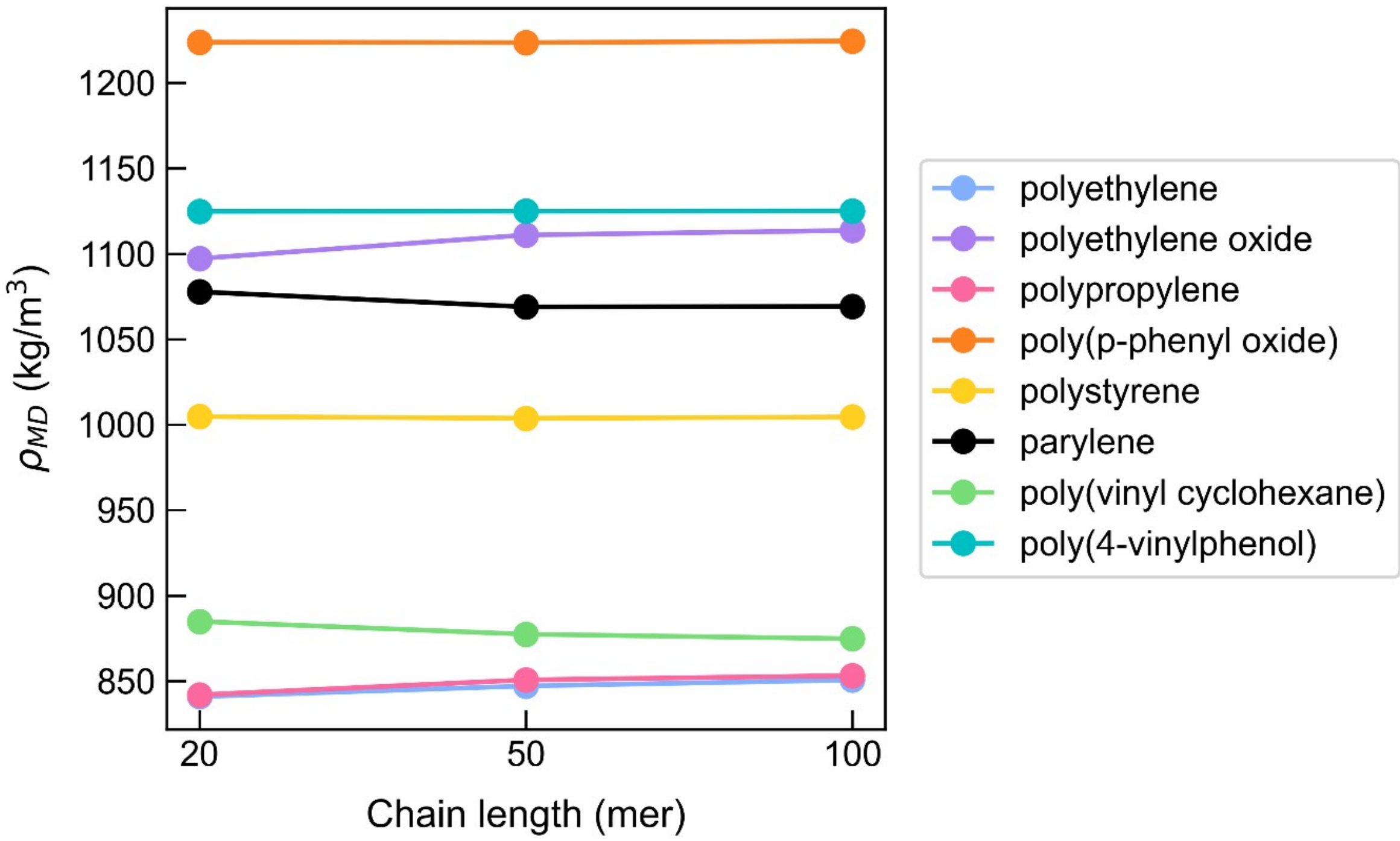

Figure S7. MD computed density, ρ_{MD}, as a function of chain length for the 8 polymers in Table S4. Standard errors are too small to display on the plot.

The box densities broadly remain consistent over each polymer length, but notably polyethylene oxide, polypropylene and polyethylene display a minor increase in ρ_{MD} from the 20- to the 50- and 100mer simulations. We attribute this to free volume effects

associated with smaller chains. In parylene and poly(vinyl cyclohexane), a slight decrease in density between 20- and 50mer is observed, which may be due differences in packing efficiency between shorter and longer chains.

To probe the structure of flexible chains further, we compute the mean square internal distances (MSID), $\langle R^2(n) \rangle$, of 20-, 50- and 100mer polyethylene, which is a well-known metric for the equilibration of polymer melts [18] and is defined as the average squared distance between polymer segments, l, separated by a contour distance, *n*. For ideal chains of sufficient length, the normalized MSID ($\langle R^2(n) \rangle / n$) should increase monotonically with *n* until a plateau value. Figure S8(a) displays this quantity for the 20-, 50- and 100mer systems after equilibration at $\Delta RDF_n <$ 0.02 was satisfied. Results were averaged over all chains and frames in the final 5 ns of the last annealing cycle. Segment separations were calculated using the first carbon of each monomer.

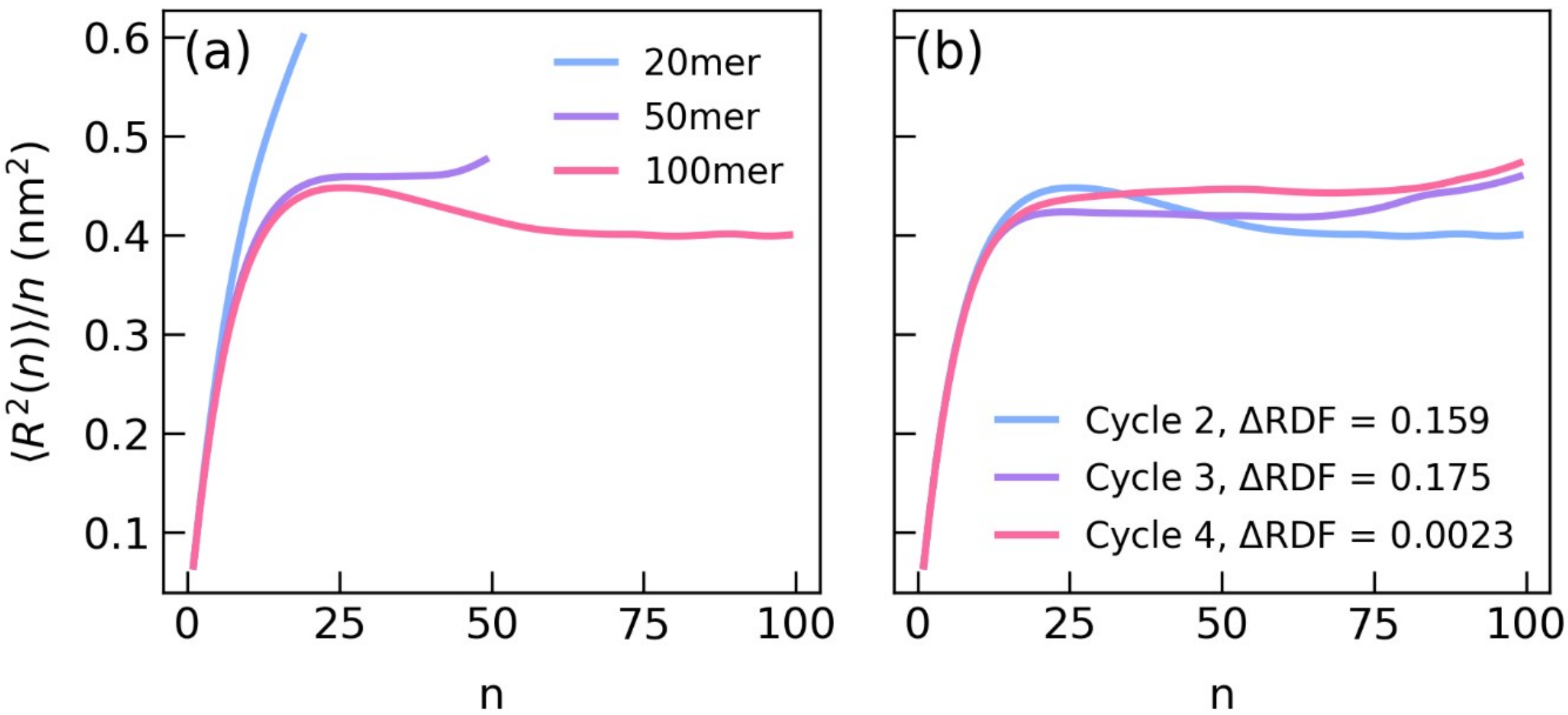

Figure S8. Normalized MSID ($\langle R^2(n) \rangle / n$) as a function of monomer separation, *n*, for (a) 20-, 50- and 100mer systems and (b) the 100mer system on annealing cycle 2, 3 and 4.

The observed continued increase in the 20mer system indicates that its conformational behaviour remains dominated by local chain stiffness. In contrast, the 50mer chain exhibits the expected MSID plateau, consistent with a relaxed chain conformation. A slight increase at large contour separations is observed for the longer chains and is attributed to finite-chain end effects and reduced statistics. The 100mer system

displays a shoulder at intermediate contour separations, indicative of residual conformational strain and incomplete equilibration after 2 annealing cycles. This feature disappears following 2 additional annealing cycles (Figure S8(b)), suggesting that higher molecular weight chains require stricter convergence criteria to achieve fully relaxed conformations.

To study equilibration in highly rigid systems of different lengths, we calculated the nematic order parameter, *S*, per annealing cycle for parylene for a maximum of 8 cycles, displayed in Figure S9.

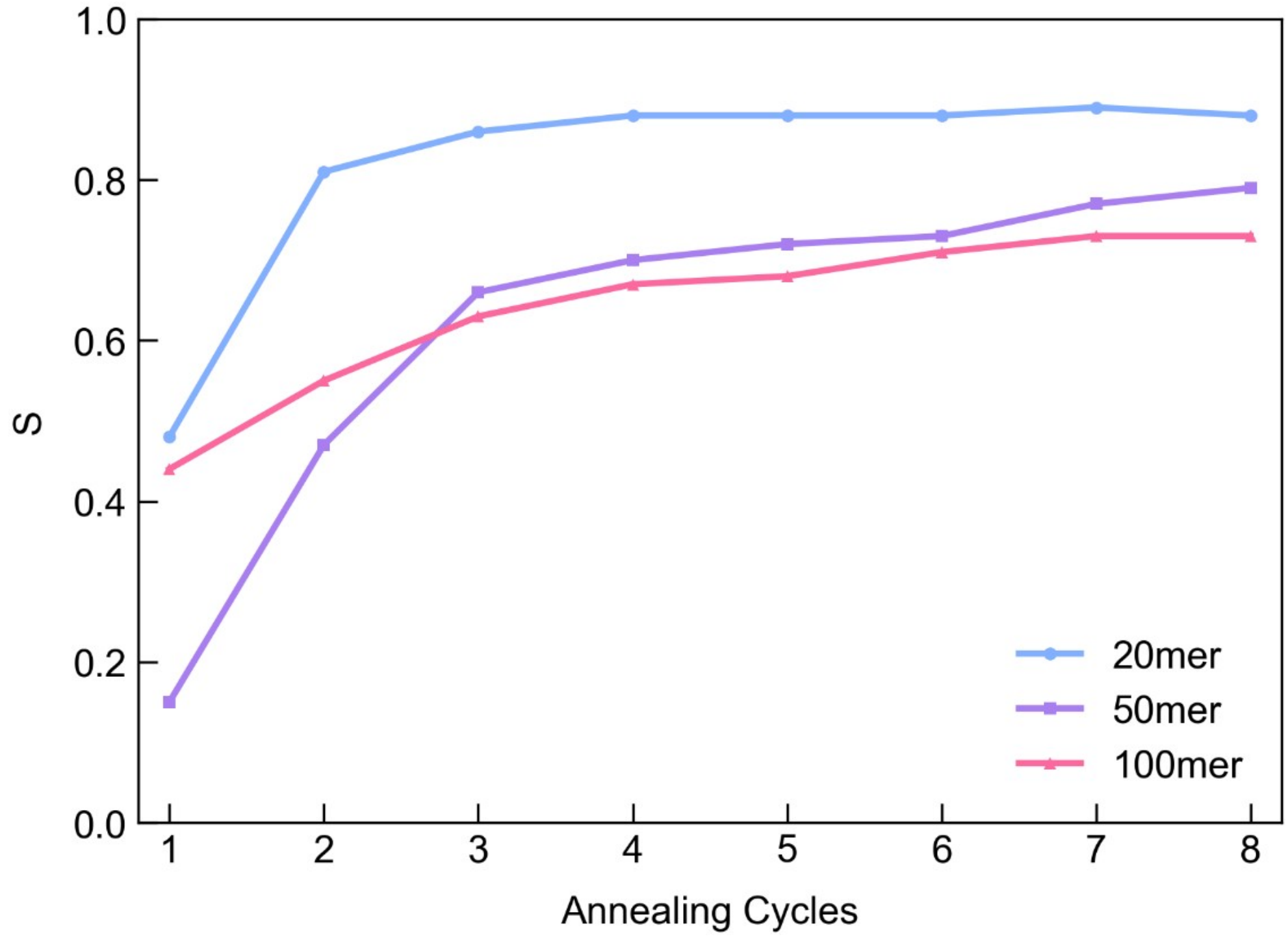

Within 4 annealing cycles, the 20mer, 100,000 atom system reaches approximately the same global order parameter value ($S = 0.88$) as in the 20mer, 50,000 atom system (see Section S.4). The 50- and 100mer systems reach lower values ($S = 0.79$ and 0.73 respectively), which may reflect reduced packing efficiency or slower equilibration in longer chains. Although all systems satisfy $\Delta RDF_n < 0.02$ after 2 annealing cycles, orientational ordering continues to evolve, indicating that this metric

at this threshold alone does not guarantee equilibrium in ordered structures. Consequently, ordering should either be monitored directly through *S* or similar metrics (see Section S.4), or the annealing protocol should be used as a preliminary screening tool.

S.7 Machine Learning Baseline Models

The baseline prediction of ρ_{MD} using the Labute approximate surface area normalised by polymer molecular weight (L-ASA/MW) is displayed in Figure S10. To simplify the model, we have used a linear regressor with leave-one-out cross validation (LOOCV).

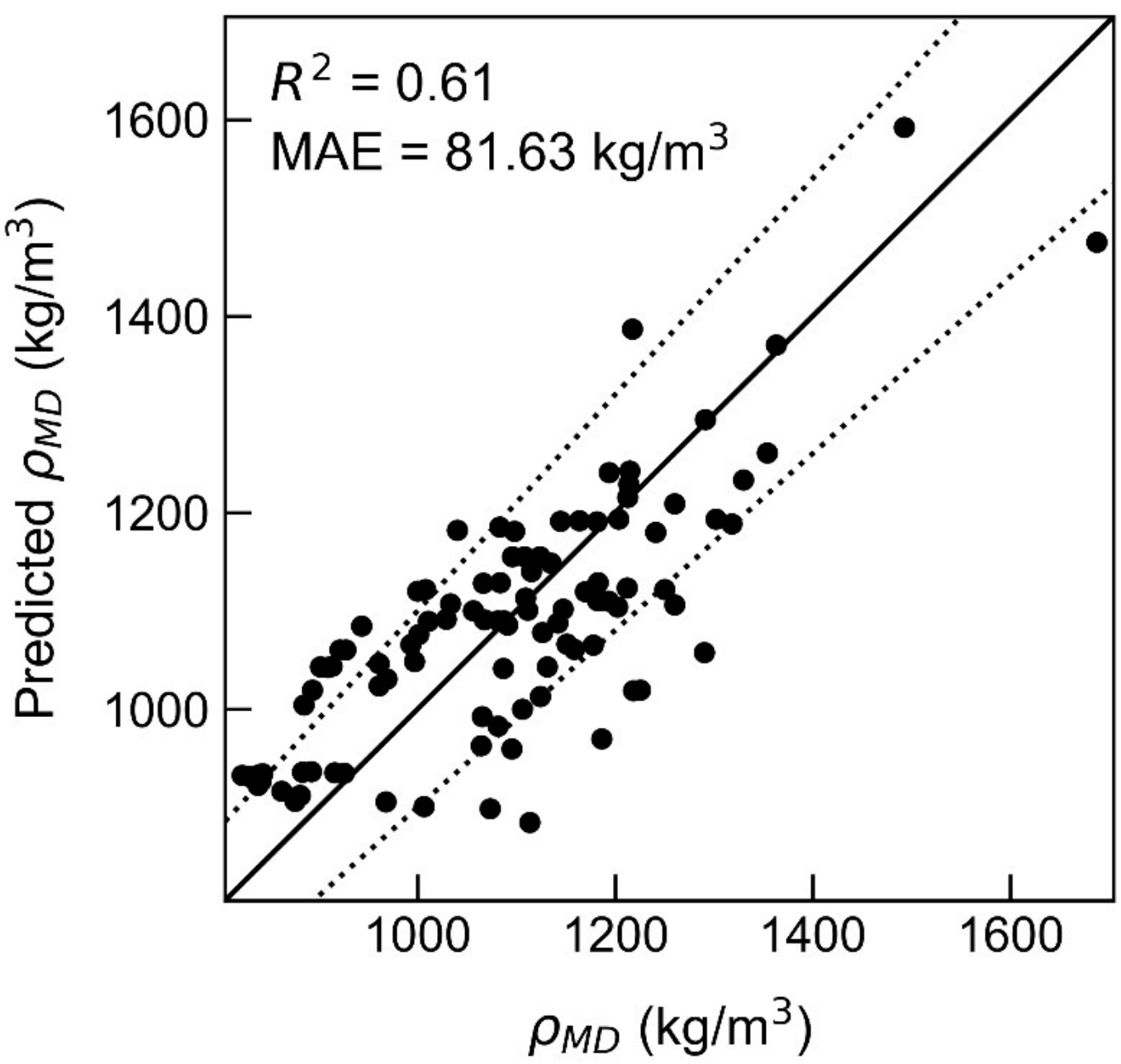

Figure S10. Predicted computed density, ρ_{MD}, against actual ρ_{MD} using a linear regression model trained with L-ASA/MW. Performance metrics are evaluated using LOOCV. Dotted lines represent the $\pm$10% error.

The prediction of T_g^{exp} using the MD-derived descriptors, T_g^{MD} and the polymer end-to-end distance normalised by its molecular weight (R_{ee}/MW) is shown in Figure S11. A linear regression model was employed to do so, and the performance evaluated with LOOCV.

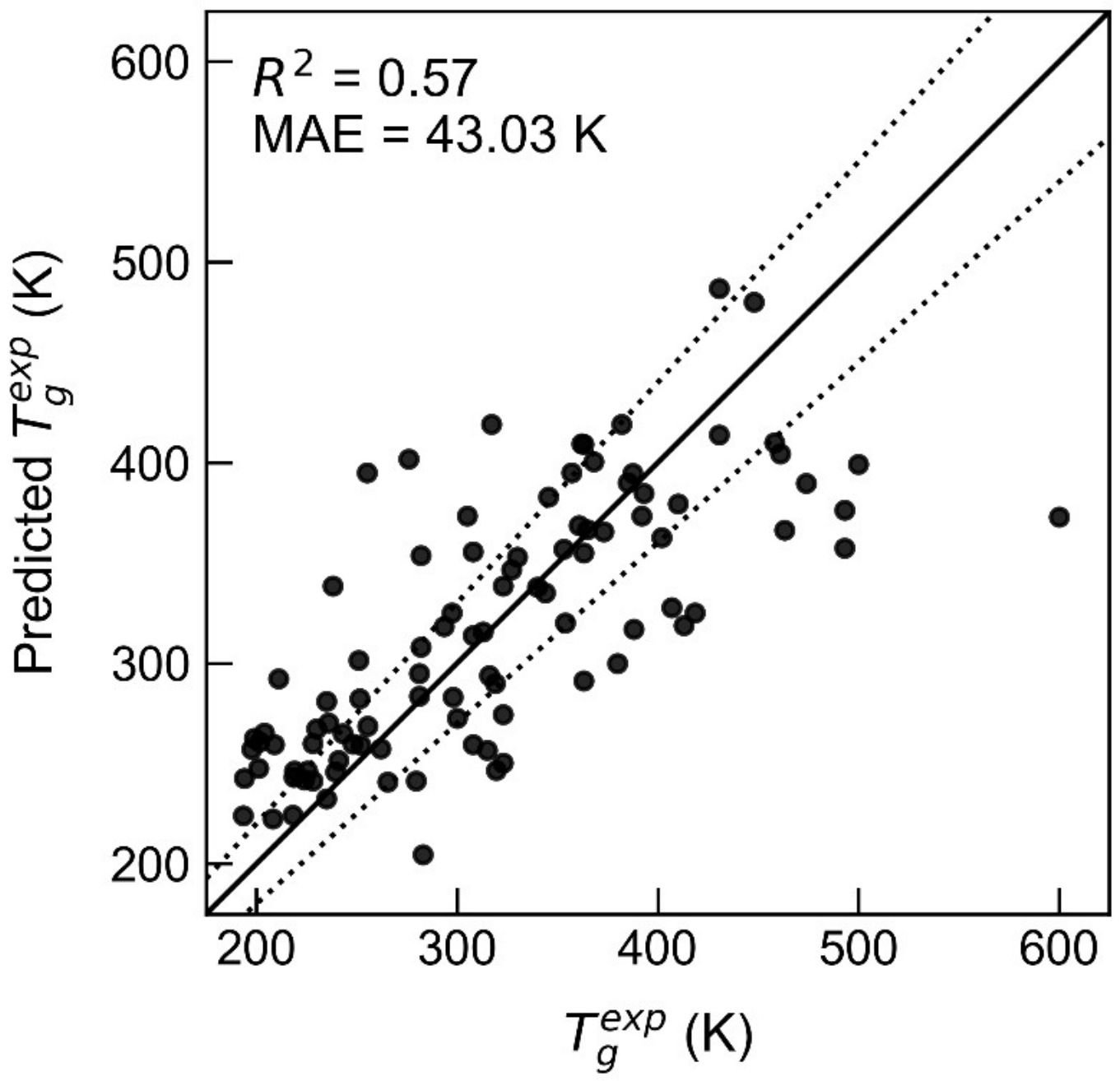

Figure S11. Predicted T_g^{exp}, against T_g^{exp} using a linear regression model trained with T_g^{MD} and R_{ee}/MW. Performance metrics are evaluated using LOOCV. Dotted lines represent the ±10% error.

S.8 Glass transition temperature

The glass transition temperature (T_g) represents a point at which polymeric systems undergo a phase transition upon cooling, from an amorphous, rubbery state to a brittle, glassy state. This change can affect the mechanical strength as well as the thermal expansion, viscosity and optical transparency. [19, 20] In polymer simulations, T_g is typically measured by calculating the change in system specific volume or density with temperature. At T_g, the specific volume exhibits a change in slope due to reduced polymer mobility. [20]

Systems that have reached equilibration within the PolyRapid framework are subjected to simulated annealing simulations from a temperature far above the T_g, 800 K to 0 K at a rate of 20 K ns^{-1}, with all other simulation conditions remaining the same as the simulated annealing step from the equilibration cycles. The density and temperature are recorded every 5 ps. We initially fit a bilinear fit across the entire temperature range following existing protocols, [21, 22] acquiring an initial value of T_g we denote as T_g^{est} within our code.

To both lower the computational expense in computing the T_g and to ensure that the computed value remains close to the experimental value we utilise our T_g^{est} value to adjust the domain over which the fitting is performed. At present we perform this by adjusting the maximum of the domain, T_{max}, as follows:

$$T_{max} = 50\left(\text{round}\left(\frac{T_g^{est} + 100}{50}\right)\right)$$

Saving between 5 and 20 ns per polymer system across the 98 polymers in this dataset by reducing the total simulation time of the heating annealing run. Upon completion, both the cooling and heating data are re-fit over 0-T_{max} computing $T_g^{cooling}$ and $T_g^{heating}$ extracting the error from the covariance matrix returned by the nonlinear least-squares optimization as implemented in SciPy. This uncertainty corresponds to one standard deviation in the fitting breakpoint temperature, cooling and heating estimates are then combined using both the arithmetic and inverse weighted arithmetic mean, with the inverse weighted mean being utilised for machine learning, taken to be the final value (T_g^{MD}). Figure S12 displays the T_g^{MD} results for our dataset compared to the available experimental values (T_g^{exp}) from various polymer handbooks and online sources. [1-3, 5-15, 23] We further exclude polymers which did not equilibrate via ΔRDF_n (see Section 3.1 of the main text), for a total of 98 polymers.

The results show a consistent overestimation in T_g^{MD} compared to T_g^{exp} with a mean absolute error (MAE) of 49-50 K, which is in-line with other MD studies calculating T_g. [24, 25]

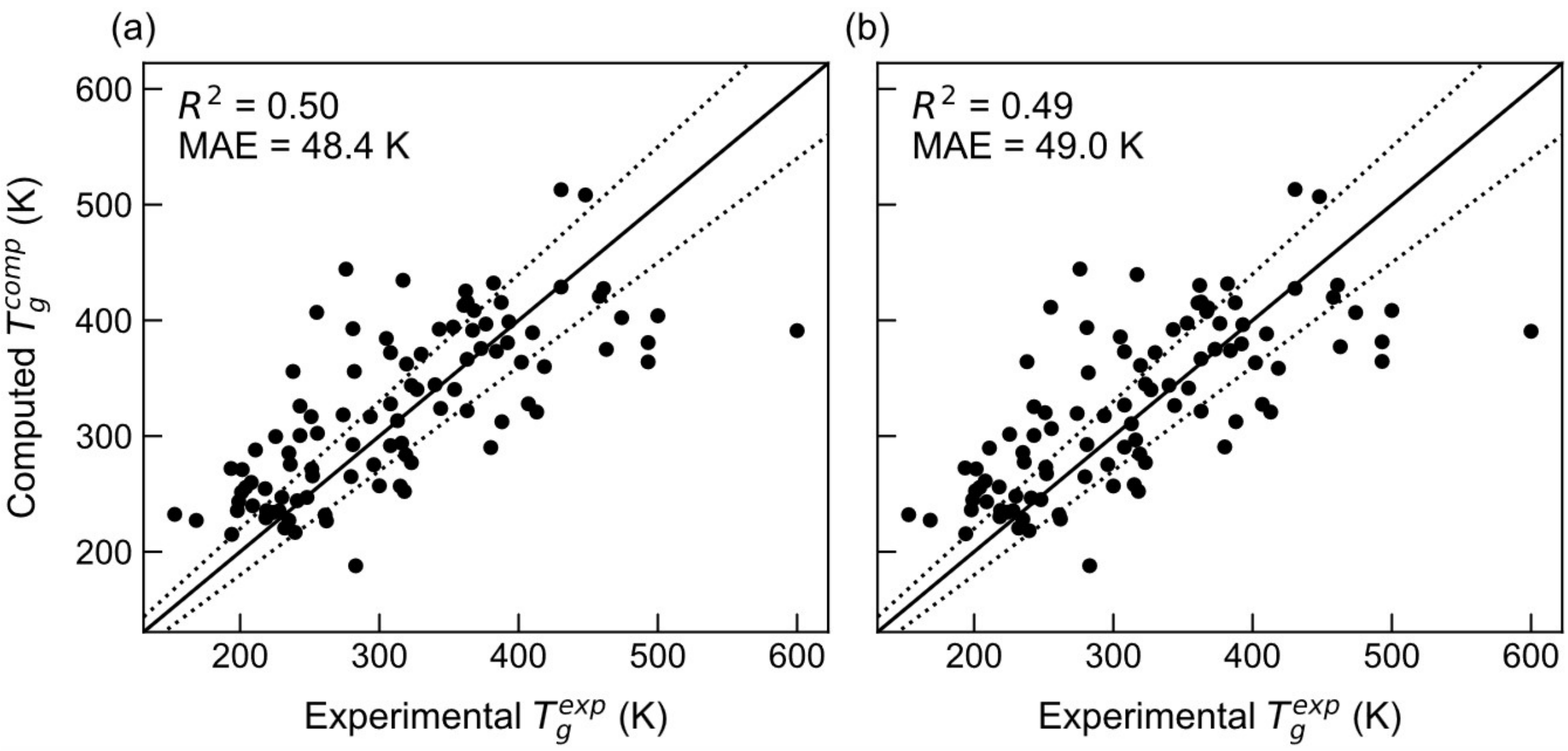

Figure S12. (a) Unweighted computed glass transition temperature, T_g^{MD} and (b) weighted computed glass transition temperature against available experimental glass transition temperatures, T_g^{exp}. Dotted lines represent the $\pm 10\%$ error.

References

1. B. Ellis and R. Smith, *Polymers: A Property Database*, CRC Press, London, England, 2008.
2. J. E. Mark, *The polymer data handbook*, Oxford University Press, New York, NY, 2009.
3. J. Bicerano, *Prediction of Polymer Properties*, CRC Press, Boca Raton, FL, 2002.

4. N,N-Dimethylacrylamide: A Versatile Monomer in Modern Chemistry, https://www.chemicalbook.com/article/n-n-dimethylacrylamide-a-versatile-monomer-in-modern-chemistry.htm, (accessed 16/04/2026, 2026.)
5. U. A. Asrorov, East Eur. J. Phys., 2024, https://doi.org/10.26565/2312-4334-2024-4-48413-418.
6. PVAM Polyvinylamine CAS 183815-54-5 Density 1.1–1.2 g/mL (25°C) for Advanced Applications, https://www.bright-chemicals.com/sale-42855319-pvam-polyvinylamine-cas-183815-54-5-density-1-1-1-2g-ml-25c-for-advanced-applications.html, (accessed 23/03/2026, 2026.)
7. 25014-15-7 (POLY(2-VINYLPYRIDINE)), https://www.chemicalbook.com/ChemicalProductProperty_EN_CB2118219.htm, (accessed 23/03/26, 2026.)
8. 25035-84-1 (POLY(VINYL PROPIONATE)), https://www.chemicalbook.com/ChemicalProductProperty_US_CB8294665.aspx, (accessed 23/03/26, 2026.)
9. J. B. Fortin and T.-M. Lu, *Chemical Vapor Deposition Polymerization: The Growth and Properties of Parylene Thin Films*, Springer US, Boston, MA, 2004.
10. Poly(N-vinyl acetamide), https://www.alfa-chemistry.com/poly-n-vinyl-acetamide-cas-28408-65-3-item-120229.htm, (accessed 23/03/2026, 2026.)
11. Poly(4-vinylphenol), Mw approx. 11,000, https://www.scientificlabs.co.uk/product/hydrophobic-polymers/436216-5G#specification, (accessed 23/03/2026, 2026.)
12. Glycidyl phenyl ether | 122-60-1, https://www.chemicalbook.com/ChemicalProductProperty_EN_CB2429443.htm, (accessed 23/03/2026, 2026.)
13. 25038-76-0 (POLY(1,3-CYCLOPENTYLENEVINYLENE)), https://amp.chemicalbook.com/ChemicalProductProperty_EN_CB1145588.htm, (accessed 23/03/2026, 2026.)
14. POLYANILINE | 25233-30-1, https://www.chemicalbook.com/ChemicalProductProperty_EN_CB7372142.htm, (accessed 23/03/2026, 2026.)
15. J. Brandrup, E. H. Immergut, E. A. Grulke, A. Abe and D. R. Bloch, John Wiley & Sons.

16. Y. Hayashi, J. Shiomi, J. Morikawa and R. Yoshida, npj Computational Materials, 2022, 8, 222.
17. G. Landrum, P. Tosco, B. Kelley, R. Rodriguez, D. Cosgrove, R. Vianello, sriniker, P. Gedeck, G. Jones, E. Kawashima, NadineSchneider, D. Nealschneider, c. tadhurst, A. Dalke, M. Swain, B. Cole, S. Turk, A. Savelev, N. Maeder, R. Walker, A. Vaucher, M. Wójcikowski, H. Faara, I. Take, V. F. Scalfani, D. Probst, K. Ujihara, Y. Pechersky, J. Monat and J. Lehtivarjo, 2026, rdkit/rdkit: 2025_09_6 (Q3 2025) Release, Zenodo.
18. R. Auhl, R. Everaers, G. S. Grest, K. Kremer and S. J. Plimpton, J. Chem. Phys., 2003, 119, 12718-12728.
19. N. Saba and M. Jawaid, Journal of Industrial and Engineering Chemistry, 2018, 67, 1-11.
20. H. Gudla and C. Zhang, J. Phys. Chem. B, 2024, 128, 10537-10540.
21. K.-H. Lin, L. Paterson, F. May and D. Andrienko, npj Computational Materials, 2021, 7, 179.
22. M. Mohammadi, H. fazli, M. karevan and J. Davoodi, European Polymer Journal, 2017, 91, 121-133.
23. C. Herfurth, P. Malo de Molina, C. Wieland, S. Rogers, M. Gradzielski and A. Laschewsky, Polymer Chemistry, 2012, 3, 1606-1617.
24. D. Martí, R. Pétuya, E. Bosoni, A.-C. Dublanchet, S. Mohr and F. Léonforte, ACS Applied Polymer Materials, 2024, 6, 4449-4461.
25. M. A. F. Afzal, A. R. Browning, A. Goldberg, M. D. Halls, J. L. Gavartin, T. Morisato, T. F. Hughes, D. J. Giesen and J. E. Goose, ACS Applied Polymer Materials, 2021, 3, 620-630.
